# Expression, Distribution and Role of Aquaporin Water Channels in Human and Animal Stomach and Intestines

**DOI:** 10.3390/ijms17091399

**Published:** 2016-08-29

**Authors:** Cui Zhu, Zhuang Chen, Zongyong Jiang

**Affiliations:** 1 Agro-Biological Gene Research Center, Guangdong Academy of Agricultural Sciences, Guangzhou 510640, China; juncy2010@gmail.com (C.Z.); chenzhuang@agrogene.ac.cn (Z.C.); 2 Institute of Animal Science, Guangdong Academy of Agricultural Sciences, Guangzhou 510640, China

**Keywords:** aquaporin, expression, distribution, stomach, intestine, physiology, pathology, cancer

## Abstract

Stomach and intestines are involved in the secretion of gastrointestinal fluids and the absorption of nutrients and fluids, which ensure normal gut functions. Aquaporin water channels (AQPs) represent a major transcellular route for water transport in the gastrointestinal tract. Until now, at least 11 AQPs (AQP1–11) have been found to be present in the stomach, small and large intestines. These AQPs are distributed in different cell types in the stomach and intestines, including gastric epithelial cells, gastric glands cells, absorptive epithelial cells (enterocytes), goblet cells and Paneth cells. AQP1 is abundantly distributed in the endothelial cells of the gastrointestinal tract. AQP3 and AQP4 are mainly distributed in the basolateral membrane of epithelial cells in the stomach and intestines. AQP7, AQP8, AQP10 and AQP11 are distributed in the apical of enterocytes in the small and large intestines. Although AQP-null mice displayed almost no phenotypes in gastrointestinal tracts, the alterations of the expression and localization of these AQPs have been shown to be associated with the pathology of gastrointestinal disorders, which suggests that AQPs play important roles serving as potential therapeutic targets. Therefore, this review provides an overview of the expression, localization and distribution of AQPs in the stomach, small and large intestine of human and animals. Furthermore, this review emphasizes the potential roles of AQPs in the physiology and pathophysiology of stomach and intestines.

## 1. Introduction

The gastrointestinal tract is the major organ for water transport and is only secondary to kidneys [1]. This fast transepithelial fluid transport occurs either via transcellular pathways, which are mediated by passive diffusion, aquaporin (AQP) water channels [2,3] and co-transporters [4,5], or via paracellular pathways by tight junctions, or both [6,7]. Among these, the regulation of AQPs driven by the osmotic forces [8] represent a major transcellular pathway for bidirectional water transport by the epithelium of the digestive tract [9,10,11].

Currently, at least 13 isoforms of AQPs (AQP0–12) have been identified in mammals. Based on their functional characteristics, they are divided into three groups. Specifically: (1) classical AQPs (AQP0, AQP1, AQP2, AQP4, AQP5, AQP6 and AQP8) are selectively permeable to water; (2) aquaglyceroporins (AQP3, AQP7, AQP9 and AQP10) are permeable to water, glycerol and urea; and (3) non-classical AQPs (AQP11 and AQP12) [12]. It has been demonstrated that AQPs are ubiquitously present in the digestive tract of mammals, including salivary gland, esophagus, stomach, small and large intestines, liver, gallbladder, bile duct and pancreas [11,13]. Water can be secreted as digestive juices and then be absorbed by the gastrointestinal epithelia [3,14], which are classified as leaky (small intestine), moderately tight (gastric antrum and colon) and tight (gastric fundus) epithelia, respectively [15]. Thus, research regarding the specific expression, distribution and roles of AQPs in stomach and intestines of human and animals has attracted much attention in the past two decades [1,3,11,16,17].

Until now, 11 isoforms of AQPs (AQP1–11) have been found to be expressed in the stomach and intestines of mammals. It is true that multiple isoforms of AQPs are commonly co-expressed in specific digestive organs or cells, and no single AQP isoform is exclusively expressed at any single site along the gastrointestinal tract [3]. In different digestive organs, AQPs have different water-transporting capacities [11]. Among these, AQP3 and AQP4 are located highly in the stomach [3,11]; moreover, AQP1, AQP3, AQP7, AQP10 and AQP11 are abundantly expressed in the small intestine, while AQP1, AQP3, AQP4 and AQP8 are the predominant isoforms in the colon [3,11].

The functions of AQPs in the stomach and intestine physiology may involve water transfer, gastric juice secretion, barrier function, as well as absorption and secretion of water and even small solutes through the epithelium [1,3,11,13]. The absorption and secretion of transepithelial fluids and solutes are closely related to gut physiology by maintaining water and electrolyte balance [1]. This balance of gut fluid movement has been indicated to be regulated by a complex of factors, including gastrointestinal hormones, intestinal contents, inflammatory factors and feeding conditions [3,18,19]. These regulations may either affect the activation or inhibition of ion and solute transport or alterations of AQPs’ expression and distribution, which may account for the changes of water transport in the gastrointestinal tract [11]. Apart from the absorption of water and small solutes, the abundant expression and localization of aquaglyceroporins (AQP3, AQP7, AQP9 and AQP10) in the gastrointestinal tracts has also been indicated to be involved in the glycerol metabolism in these tissues [20]. Previous studies have demonstrated that the alterations of AQPs expression and distribution in the gastrointestinal tracts are associated with gut disorders and diseases [1,21,22]. Thus, this review mainly summarizes recent advances in the expression, localization and distribution of AQPs in the stomach and intestine of human and animals and emphasizes the potential roles of AQPs in gut physiology and pathophysiology.

## 2. Expression, Distribution and Role of AQPs in the Stomach of Humans and Murine Rodents

### 2.1. Normal Physiological Conditions

#### 2.1.1. Human Stomach

Stomach exhibits its major functions via the secretion of gastric acid, enzymes and hormones, which are essential for nutrients’ digestive and absorptive processes [23]. The secretion of isotonic hydrochloric acid (HCl) by parietal cells of gastric glands is usually accompanied by water transport from the membrane [3]. Due to the relatively low permeability of gastric parietal cells to water and small solutes [24], the paracellular pathway has been proposed as the major water transport route in gastric juice production for years. However, the expression of AQPs in the stomach may indicate their important roles in the regulation of stomach physiology.

Currently, at least ten isoforms of AQPs (including AQP1, AQP2, AQP3, AQP4, AQP5, AQP7, AQP8, AQP9, AQP10 and AQP11) have been identified to be expressed in human stomach (Table 1). Notably, the mRNAs of the AQP1, AQP3, AQP4, AQP5 and AQP11 isoforms are expressed in human normal gastric tissues [25]. Additionally, AQP7, AQP8 and AQP10 mRNAs have been reported in the body and pyloric antrum of healthy human stomach [3]. However, the mRNAs of other AQP isoforms (AQP0, AQP6 and AQP12) were absent in different portions of human stomach [3,25]. Even though the presence of AQP1 protein expression was not confirmed in human stomach epithelia and mucosa by immunohistochemistry, AQP1 was detected at a low expression level in the endothelial barriers of the human gastrointestinal tract, including gastric antral and oxyntic mucosa using tissue microarrays [26]. Moderate and low expression of AQP2 was found in human antral and oxyntic gastric mucosa using tissue microarrays, respectively [27]; further immunohistochemistry analysis showed that human AQP2 was localized to the gastric pits [27]. Moreover, AQP4 protein is mainly localized to the basolateral membrane of parietal cells and chief cells in the normal human gastric mucosa [25,28], indicating their possible roles in gastric acid and enzyme secretions. The AQP3 and AQP5 localizations were also detected in the human gastric mucosa by immunofluorescence [25]. However, as mentioned above, despite the presence of AQP1, AQP7, AQP8, AQP10 and AQP11 mRNAs in human stomach [3,25,26], their protein expression and localization remains to be determined (Table 2).

#### 2.1.2. Murine Stomach

As shown in Table 1, at least six isoforms of AQPs (AQP1, AQP3, AQP4, AQP5, AQP6 and AQP8) are present in the stomach mucosa of murine rodents, as demonstrated by the methods of reverse transcription-polymerase chain reaction (RT-PCR), Western blotting, ELISA, in situ hybridization, immunofluorescence and immunohistochemistry [18,19,31,32,33,34,35,36,37,38]. These AQPs are mainly present in the gastric mucosa and glands, and they have been mostly reported in rats (Table 2). The presence of these AQPs in glandular stomach may indicate their roles in gastric secretion. For example, AQP1 protein was shown to be expressed in the mucosa surface of rat stomach [31]. By using immunohistochemistry, AQP3 protein was detected in the basolateral membrane of glandular stomach [32]. Moreover, AQP4 expression has been demonstrated in the stomach of rats, mice and guinea pigs, but with different distributed localizations [31,33,34,35,36,37]. In Sprague-Dawley rats, AQP4 protein is expressed not only in gastric mucosa [31], but also in the basolateral membranes of mature parietal cells in specific locations along the axis of the gastric glands [34]. In mice, AQP4-positive parietal cells are distributed in the basal side of gastric fundic mucosa [35], especially in the basolateral membrane of parietal cells [36,37]. AQP4 protein is expressed in parietal cells of the stomach in guinea pig [33]. However, the gastric fluid secretion and gastric pH were not affected in the AQP4-knockout mice compared to the wide-type mice [36]. Furthermore, the expression of AQP6 at both mRNA and protein levels and its distribution were demonstrated in the rat stomach [19]. Using in situ hybridization, AQP6 mRNA was determined to be present in the isthmus, neck and basal region of the rat stomach; AQP6 immunolabeling was confirmed in the isthmus, neck and some parietal cells of the rat stomach using immunohistochemistry [19]. Neither AQP7 mRNA expression nor AQP7 protein expression were detected in the rat stomach [14]. However, only a low mRNA level of AQP8 in the forestomach and glandular stomach of rats was demonstrated by RT-PCR, while its protein expression or immunolocalization could be not be detected [32].

### 2.2. Pathological States

#### 2.2.1. Chronic Gastritis

Chronic gastritis remains one of the most common serious pandemic infections affecting men and women, which can lead to gastric ulcers and gastric cancers [45]. Several AQPs have been found in chronic gastritis. For example, AQP3 mRNA is expressed in chronic atrophic and chronic superficial gastritis patients [46]. There is a relationship between the altered human AQP3 and AQP4 mRNA expression in the mucosa of upper stomach, and the degrees (severe, mild or normal control) of spleen-stomach dampness-heat syndrome in human chronic superficial gastritis, with higher gene expression of AQP3 and AQP4, were observed in the moderate and severe groups compared with the other two groups [30]. In addition, a correlation between AQP3/AQP4 expression and gastritis types was proposed [47]. A significantly higher expression of gastric mucosal AQP3 was detected in patients with chronic superficial gastritis compared with patients with chronic atrophic gastritis [46]. Besides, AQP3 and other AQPs (AQP5, AQP7 and AQP11) were also upregulated at the mRNA level in atrophic gastritis [3]. Bodis et al. (2001) have observed an increased expression of both AQP1 and AQP4 in rats with gastritis, but without any macroscopically-detectable changes in the stomach [31].

*Helicobacter pylori* infection is the major cause for chronic gastritis. It has been proposed that *Helicobacter pylori* are essential for the development of gastric tumorigenesis [48]. A recent study has suggested that AQP3 is involved in *Helicobacter pylori* infection-related gastric carcinogenesis since AQP3 expression was upregulated both in human gastric adenocarcinoma cells and in rat gastric tissues [49]. However, in the *Helicobacter pylori*-infected mice, the ratio between AQP4 and H^+^/K^+^ATPase mRNA expression was significantly decreased in histamine type 2 receptor knockout mice [35]. Notably, the protective effect of calcitonin gene-related peptide on gastric mucosa injury in rats was demonstrated to be involved in inhibition of AQP4 expression and mast cell degranulation and in regulation of several hormone genes’ expression (such as gastrin and somatostatin) [50]. Additionally, another study suggested that both AQP1 and AQP4 were important for the maintenance of mucosal integrity, and their expression in the stomach was increased inethanol-induced edema and after gastric injury [31]. Collectively, these results indicate that the expression of several subtypes of AQPs (AQP1, AQP3, AQP4, AQP5, AQP7 and AQP11) is upregulated by gastritis, and more studies are essential to investigate their potentials as diagnostic biomarkers and drug targets for gastritis therapy.

#### 2.2.2. Gastric Cancer

Gastric cancer is one of the most common cancers worldwide. It remains a major cause of mortality and morbidity [51]. Evidence has shown that various types of ion channels, water channels and pH regulators were expressed in gastric cancer cells and tissues, and changes in their expression and activities may be involved in the pathology and development of gastric cancer [52,53]. The roles of AQPs in gastrointestinal malignancies have been summarized in a recent review [52]. Evidently, increasing studies have shown that AQPs behave more than just as water channels in the body, but may be involved in migration, proliferation, adhesion and angiogenesis [54,55,56]. Several AQPs have been reported to be expressed in gastric cancers, which indicated their potential involvement in human gastric carcinogenesis. For example, the mRNAs of AQP1, AQP3, AQP4, AQP5 and AQP11 are also found in human gastric cancers [25]. AQP1 has been demonstrated to play a crucial role in cell migration of rat gastric epithelial cell line during wound healing [57]. The knock down of AQP1 by siRNA resulted in a marked delay of wound healing [57]. Moreover, Watanabe et al. (2009) has reported that AQP5 protein is localized in the apical membrane of the human gastric cancer cells and the human gastric adenocarcinoma cell line [29]. AQP5 promotes the proliferation and migration of human gastric carcinoma cells, and its overexpression was correlated with enhanced lymph node metastasis [58]. The increased expression of AQP5 may indicate its role in the cell differentiation of human gastric adenocarcinomas [29]. In addition, AQP3, AQP4 and AQP5 exhibited differential expression between human gastric carcinomas and corresponding normal tissues; AQP3 and AQP5 protein expression was detected as remarkably stronger in the human carcinoma tissues than that in normal mucosa by immunofluorescence [25]. In contrast, AQP4 was found to be absent in human carcinoma tissue in contrast with healthy tissue [25], indicating its downregulation during gastric tumorigenesis. Interestingly, AQP9 facilitated water fluxes for epithelial wound healing based on its involvement in the migration of monolayer epithelial cells [59].

Undoubtedly, AQP3 was best studied and was believed to be essential for gastric tumor growth and spreading. AQP3 expression is higher in human gastric cancer tissue compared with that in normal tissue, as well as in the human gastric carcinoma cell lines by Western blotting analysis [60]. The involvement of AQP3 in carcinogenesis and the progression of gastric carcinoma can be due to upregulation of AQP3, which promotes the proliferation, migration and invasion of human gastric carcinoma cells [56,61]. It seemed that both the ERK and PI3K/AKT signaling pathways were involved in the upregulation of AQP3 expression, which was induced by hyperglycemia in human gastric carcinoma [62]. Similarly, another study pointed out that c-Met could regulate the AQP3 expression via the ERK signal pathway in human gastric carcinoma, which affected the metastasis and invasion of human gastric carcinoma [63]. Huang et al. (2010) has also demonstrated that AQP3 played a critical role in human epidermal growth factor (EGF)-induced migration of human gastric cancer cells via ERK signal transduction pathways [60]. Additionally, a recent study has shown that AQP3 promotes the tumorigenic potential of several gastric cancer cell lines by activating the Wnt/GSK-3β/β-catenin pathway [56]. Moreover, in a recent study, AQP3 has been proposed as a potential biomarker for the diagnosis of gastric intestinal metaplasia, which may finally transform into gastric cancer [64].

Collectively, AQPs-dependent cell migration has significant implications in tumor metastasis and wound healing, since AQPs facilitate the cell shape changes and propel the cell forward by changing the cell volume with water flow regulation [65]. Additional studies are required to determine whether these AQPs can become potential therapeutic targets for gastric cancers.

## 3. Expression, Distribution and Role of AQPs in the Intestines of Humans and Murine Rodents

### 3.1. Normal Physiological States

#### 3.1.1. Human Small Intestine

Small intestine (duodenum, jejunum and ileum) is the major site for digestion and absorption of nutrients and water. For human, the daily fluid transport in small intestine is approximately 9 L, which is composed of about 2 L of water intake and about 7 L of secretion of digestive juices [1]. The rapid bidirectional movement of this large volume of water, which involves both absorptive and secretory processes, largely depends on enterocytes [3]. The expression of AQPs in the human and murine small intestine is summarized in Table 3. At least eight isoforms of AQPs (AQP1, AQP2, AQP3, AQP4, AQP7, AQP8, AQP10 and AQP11) are present in human small intestine. A previous study has demonstrated that AQP1, AQP3 and AQP4 mRNAs are expressed in human small intestine, without any detectable mRNAs of AQP0, AQP2 and AQP5 [66]. However, another study by tissue microarrays has shown the very weak expression of AQP2 in human small intestinal mucosa [27]. Moreover, AQP3 protein expression was confirmed in human small intestine via Northern blotting [67]. In addition to AQP1 and AQP3 as mentioned above, the AQP7 and AQP8 expression at both mRNA and protein levels was also demonstrated in the mucosal epithelium of human ileum [21]. Moreover, abundant AQP11 mRNA has been confirmed in the healthy human duodenum tissues [3]. Notably, the aforementioned AQPs are mainly distributed in the villi and crypt of the enterocytes in human small intestine (Table 2). Evidently, AQP1 protein is widely expressed in the human small intestinal capillary endothelia and erythrocytes, but not in the mucosal epithelium [21,39]. However, the immunostaining of AQP3 and AQP8 was strongly detected along the apical parts of the ileum surface epithelium (enterocytes), while AQP7 immunoreactivity was mainly found in the basolateral parts of surface epithelia in the human ileum by immunofluorescence [21]. AQP3 was also found to be expressed highly in basolateral membranes of villi epithelial cells and goblet cells, as well as Paneth cells and granule-containing cells in human ileum crypts [39]. Besides, AQP10 was found to be expressed abundantly in the absorptive epithelial cells of human small intestine [39,68,69], with much higher expression in duodenum and jejunum than in ileum [68,69]. Moreover, AQP10 is expressed highly in the apical membrane of goblet cells in human ileum villi [39]. These results suggest that AQP1, AQP3, AQP7, AQP8 and AQP10 are the predominant AQPs in the human small intestine [21,39]. The expression of AQPs in the superficial villi and the crypt of enterocytes otherwise indicates their roles in absorption and secretion, respectively [3]. Although the presence of AQP4 and AQP11 mRNAs was demonstrated as mentioned above, their protein expression and localization in human small intestine remain to be investigated.

#### 3.1.2. Murine Small Intestine

As shown in Table 3, there are at least nine isoforms of AQPs (AQP1, AQP3, AQP4, AQP5, AQP6, AQP7, AQP8, AQP9 and AQP11) present in the small intestine of murine rodents, including rats, mice and guinea pigs. A previous study has shown that the expression of AQP1, AQP3 and AQP4 was present in the small intestine of rats [70]. On the other hand, AQP1, AQP3, AQP7 and AQP11 mRNAs were demonstrated to be expressed in the enterocytes of jejunum and ileum of mice [71]. Both nonglycosylated and glycosylated forms of AQP1 were found to be localized in the apical and basolateral membranes of cells of Brunner’s gland in the duodenum of rats [18]. Besides, AQP1, first known as CHIP, has been shown to be abundantly expressed in the rat duodenal and ideal lacteals [72]. Notably, the AQP3 mRNA level is increased along the length of the intestine in rats, with abundant expression in the distal ileum [73]. Using immunohistochemistry, AQP3 staining is confined to the basolateral membrane of absorptive epithelial cells lining the lumen and to the neck of crypts [32]. The AQP3 and AQP8 proteins are localized to the basolateral membranes of rat ileum and to the subapical compartment of epithelial cells of jejunum and ileum, respectively [32].

Notably, AQP4 was expressed in both rats and guinea pigs, with its expression in the absorptive and glandular epithelial cells of small and large intestine [33], as well as in the enteric glial cells of guinea pig [33]. In addition to the AQP5 expression at both mRNA and protein levels, AQP5 localization was also demonstrated in the apical and lateral membranes of the pyloric gland of the stomach, as well as in Brunner’s gland of duodenum in rats [18,38]. However, no AQP5 expression was detected in the intestinal glands or cells in the villi of rat duodenum [76].

A previous study has demonstrated the distribution and expression of AQP6 along the rat small intestine, at both mRNA and protein levels [19]. Specifically, the jejunal AQP6 mRNA expression was increased after feeding, indicating its direct involvement in the absorption of water and anions [19]. The gastrointestinal fluid recirculation is significantly increased during a meal to facilitate proper digestion and absorption of intestinal contents [11], which leads to possible alterations of AQPs’ expression thereafter.

The expression of AQP7 at both mRNA and protein levels has been found to be abundant in the rat small intestine [14]. There is a much stronger expression in the surface epithelial cells of the duodenum, jejunum and ileum when compared to the crypt cells at the basolateral side [14]. Further studies have confirmed the mRNA and protein expression of AQP7 and AQP8 in the apical brush border membrane of epithelial cells in the rat small intestine [75].

In addition, AQP8 has been shown to be present in the surface epithelial cells in the duodenum and jejunum of rats [74]. Elkjaer et al. (2001) found that AQP8 mRNA was detected in the duodenum and proximal jejunum [77]. Interestingly, AQP9 mRNA and protein expression, otherwise, has been confirmed in the mucus-secreting goblet cells of mice duodenum, jejunum and ileum, which indicated the potential role of AQP9 in mucus secretion for protecting the intestine from pathogen invasions [40].

#### 3.1.3. Human Large Intestine

Large intestine, mainly referring to the colon, extracts water (approximately 2 L daily) and electrolytes from the solid wastes to produce dehydrated feces [1]. Indeed, it has been proposed that water movement in this site may be mediated mainly through AQPs expressed in the colon [3]. The expression of AQPs in the human and murine large intestine is summarized in Table 4. There are at least eight isoforms of AQPs (AQP1, AQP2, AQP3, AQP4, AQP7, AQP8, AQP10 and AQP11) expressed in human large intestine, and most have been reported to be in the colon [3,8,21,27,42,44]. As shown previously, AQP1, AQP3, AQP7 and AQP8 are the predominant AQPs in the human colon [21]. Besides, AQP2 was found to be expressed at a low level in the colonic mucosa by human tissue microarrays and immunohistochemistry [27]. AQP3 is also expressed in the human intestinal epithelial cell line (HT-29) [78]. For localization determination, AQP3 was found to be distributed in the apical membrane of villus epithelial cells in the human colon [21,42]. In other studies, it has been indicated that AQP3 is selectively targeted to the basolateral membrane of the human colon [43]. AQP4 mRNA was also detected at a very low level as shown by RT-PCR in human normal colons, but its specific staining was not found by immunofluorescence [44]. Moreover, AQP7 immunoreactivity was detected in the basolateral epithelia in the villi and crypt of human colon [21]. AQP8 is expressed in the apical sides of the villus and crypt epithelial cells in human colon [8,21]. Another study has also shown that both AQP7 and AQP8 are mainly expressed in the apical parts of human colonic tissues, with some cytoplasmic and basolateral distributions [44]. Moreover, AQP10 mRNA is expressed at a very low level in the ascending colon of human, while AQP11 mRNA is abundantly present in human ascending colon [3].

#### 3.1.4. Murine Large Intestine

As listed in Table 4, there are at least nine isoforms of AQPs (AQP1, AQP2, AQP3, AQP4, AQP6, AQP7, AQP8, AQP9 and AQP11) that are expressed in the large intestine of murine rodents. Specifically, AQP1, AQP4, AQP8 and AQP11 mRNAs were all detected in the mice colons [71]. Another study also demonstrated the mRNA and protein expression of AQP4, AQP7 and AQP8 in mice colons [44]. Previous studies have shown that AQP3 was expressed in the basolateral membranes of the epithelium in rats colons [32] and in mice [78]. However, the expression of AQP3 in the colon and ileum was greater than in the stomach [80], and AQP3 has been believed to be a dominant AQP subtype in the rat colon [82]. Additionally, using semi-quantitative RT-PCR and immunoblotting, the AQP3, AQP4 and AQP8 expression was clearly shown in the apical membrane of superficial colonocytes in rats [81]. Thus, AQP3 has been shown to be distributed in both the apical and lateral colonocyte membranes in addition to staining a perinuclear region [83]. Gallardo et al. (2001) have demonstrated that AQP2 is localized to the apical membrane of surface absorptive epithelial cells in the rat distal colon, indicating the role of AQP2 in water absorption in the colon [41]. Moreover, AQP4 is immunolocalized to the basolateral membrane of colonic surface epithelium of wide-type mice, but not in AQP4 knockout mice [84]. The distribution and expression of AQP6 at both mRNA and protein levels was demonstrated in rat colon and cecum, and its expression was higher in the large intestine than the small intestine [19]. In contrast to the small intestine, AQP7 expression was found to be weaker in the large intestine of rats [14]. Apart from the duodenum and jejunum, AQP8 was also present in the surface epithelial cells in the colon of rats [74]. AQP8 mRNA was detected in the proximal colon and rectum of rats, with protein immunolocalization specifically in the absorptive epithelial cells of these intestinal segments [77] and mainly localized to the subapical compartment of intestinal epithelial cells [32], which indicated its possible roles in the secretion or absorption of water at these sites [85]. The presence of AQP9 mRNA has also been demonstrated to be exclusively localized in goblet cells at the bottom of crypts in the mouse colon, as determined using in situ hybridization [40]. Moreover, AQP1 was also demonstrated in the rat colon lymphatic lacteals [72], and AQP4 was found to be expressed in the colonic enteric neurons of rats and mice [86].

### 3.2. Pathological States

#### 3.2.1. Enteritis

Enteritis is characterized by abnormal electrolyte and water transport events that are accompanied by intestinal inflammation and injury [44,87]. The reduced mRNA expression of several AQPs (AQP1, AQP3, AQP7 and AQP8) in human intestinal mucosa has been demonstrated at the early stage of inflammatory bowel diseases (IBD), including Crohn’s disease and ulcerative colitis [21]. AQP4, AQP7 and AQP8 mRNAs were all detected in both normal colons and in the colons of patients with ulcerative colitis, Crohn’s disease and infectious colitis [44]. In the trinitro-benzene-sulfonic acid-induced colitis of rats, the mRNA and protein expressions of AQP3 and AQP8 were also downregulated in the ileum and colon [87]. This suggests that AQP3 and AQP8 may play significant roles in the regulation of intestinal fluid homeostasis and disorders in rats [32,87]. Indeed, the AQP3-null mice displayed impaired enterocyte proliferation and developed severe colitis after dextran sulfate treatment, probably due to the impaired glycerol-transporting function [88]. Moreover, the abnormal regulation of fluid and electrolyte flux in IBD patients and in a murine colitis model may be associated with significant reductions in AQP4 and AQP8 mRNA expression in the colon [44]. Accordingly, apical AQP8 immunolabeling was reduced in the colon of IBD patients, either in the surface epithelium or in the crypts [21]. The inhibition of AQP8 expression by siRNA significantly decreased the osmotic water permeability in isolated superficial colonocytes in the rat proximal colon [81]. These results may suggest that the role of AQP8 in osmoregulation and mucosal fluid fluxes should be elucidated [77]. Notably, heat stroke has been shown to induce jejunum barrier damage and cell apoptosis via the increased expression of AQP1, AQP3, AQP7, AQP8 and AQP11 mRNA [89]. These results collectively indicated that enteritis, either in murine rodents or human, may be associated with alterations in electrolyte and water transport mediated by a downregulation in AQPs’ expression, especially for AQP3, AQP4 and AQP8. Moreover, it has been indicated that the structural changes of AQP3 and AQP10 in human intestine may lead to fluid imbalance, thus facilitating the development of Crohn’s disease and ulcerative colitis [43]. Notably, pro-inflammatory cytokine TNF-α has been shown to decrease AQP5 protein and mRNA expression in a mouse lung epithelial cell line [90]. However, it is still not clear whether the inflammatory cytokines released during intestinal inflammation would exacerbate abnormal electrolytes and water transport due to the alterations of AQPs expression or distribution. Thus, further experiments should be conducted.

#### 3.2.2. Diarrhea

The defects and disorders of secretion and water absorption in the intestines are important factors in the pathogenesis of diarrhea [71]. During infectious diarrhea, the rapid loss of fluids and electrolytes is involved in this process due to increased intestinal secretion and/or decreased intestinal absorption [91]. Water channel proteins may otherwise be involved in the normal dehydration of fecal contents; thus, changes in the distribution of AQPs may play an important role in the development of diarrhea [83]. 

Changes in the expression or distributions of AQPs in the development of several types of diarrhea in human and animals are summarized in Table 5. Clearly, several AQPs have been demonstrated to be downregulated under different types of diarrhea [22,71,93,95,96,97,98,100]. Moreover, the exposure to mercury, which inhibits of AQPs, significantly downregulated gastric AQP3 and AQP4 mRNA and their protein expression, as well as the AQP3 and AQP7 protein expression in the small and large intestines of rats, thus leading to the accumulation of intestinal fluids and, finally, diarrhea [101]. These results suggested that these AQPs may be potential targets for the prevention and treatment of diarrhea in human and animals. Increasing evidence has demonstrated that the anti-diarrhea effects of some substances, including emodin [78] and berberine [102], and the laxative drugs magnesium sulfate (MgSO_4_) [82,103], may involve the alteration of AQPs to regulate water transport and absorption possibly, via the regulation of the cAMP-dependent PKA/p-CREB signal pathway [78,82,103]. A recent study has also shown that the upregulation of AQP2 in the distal colon was found in cirrhotic rats with ascites, and its expression is inhibited by Tolvaptan, which probably leads to decreased water reabsorption and induces diarrhea in cirrhotic rats with ascites [79].

Notably, the importance and functions of AQP water channels in fluid transport were not confirmed by AQP-null mice, and surprisingly, an apparent phenotypic abnormality is not associated with the tissue-specific expression of AQPs [104]. For example, a previous study using AQP4 knockout mice indicated that transcellular water transport through AQP4 facilitates transepithelial osmotic water permeability, but it marginally affects colonic fluid secretion or fecal dehydration [84]. Moreover, the AQP1-null mice displayed defective fat processing, which resulted in an increased stool fat content, whereas AQP4 knockout mice exhibited defective fecal dehydration and colonic fluid absorption compared to those of wild-type mice [1].

#### 3.2.3. Colon Cancer and Rectal Cancer

Previous studies have shown that the alterations of the expression and localization of several AQPs (such as AQP1, AQP3, AQP5 and AQP8) in the colon and rectum may imply their involvements in the development of colon and rectal cancers [52], probably due to their roles in cell migration, proliferation and angiogenesis, as mentioned above. Specially, AQP1, AQP3 and AQP5 expression has been demonstrated in seven human colon and colorectal cancer cell lines, and they are associated with an early stage of colorectal cancer development [105]. Additionally, human epidermal growth factor significantly increased the expression of AQP3 and consequently the migration of human colorectal carcinoma cells (HCT116) in a dose- and time-dependent manner [106]. These data suggested that the overexpression of AQP3 may facilitate the migration of colorectal carcinoma cells [106]. Interestingly, it has been shown that AQP3 may impair the intestinal barrier integrity [107], which is probably mediated by miR-874 through targeting AQP3 following intestinal ischemic injury [108]. It is still unknown if the regulation of AQP3 in intestinal barrier functions is associated with the progression of intestinal cancers, and it requires further study. There was no expression of AQP8 in human colorectal tumors, which indicated a downregulation of the *AQP8* gene during tumorigenesis [8].

## 4. Expression and Distributions of AQPs in the Stomach and Intestines of Domestic Animals

In contrast to the findings in humans, rats and mice, there are limited reports regarding the expression of water channel proteins in domestic animals. The water absorption along the large intestines of pigs is lower than those of cattle and sheep, probably due to the difference in the rates of passage of digesta among these species [109]. Previous studies have demonstrated the expression and localization of AQP1, AQP4 and AQP5 in both the small intestine and large intestine of colostrum-suckling buffalo calves [110,111,112]. Specifically, AQP1 immunoreactivity is abundant in the endothelium and is moderate in the enterocytes of small and large intestines in newborn buffalo calves after suckling colostrum for seven days [111]. AQP4 is mainly distributed along the endothelium and enterocytes, whereas AQP5 is mainly in the endocrine cells of the large intestine [110,112]. Moreover, the AQP3, AQP7 and AQP10 transcripts, together with the protein abundance of AQP3, AQP7 and AQP8, have also been confirmed in ruminal papillae of lactating dairy cows [113]. Interestingly, AQP10 mRNA in the duodenum and jejunum of cattle could not be detected by RT-PCR due to the fact that AQP10 is a pseudogene in cattle and in their relatives (sheep and goat) [114].

However, pigs are recognized as the best research model for humans due to their similarities in gut structure and physiology. Importantly, AQP1 was found to be distributed extensively in the epithelium and endothelium of the gastrointestinal system of pigs [115]; however, the role of AQP1 in the fluid secretion, absorption and pathophysiology of the porcine gastrointestinal system remained controversial [116]. The expression of AQP1 protein was also demonstrated in the enteric nervous system of the ovine duodenum, with predominant distribution in the sensory submucosal neurons instead of the myenteric neurons [117]. In a microarray analysis, AQP4 mRNA has been detected in both the oxyntic and pyloric mucosa of weaned pigs, but with higher expression of AQP4 in the oxyntic mucosa related to hydrochloric acid and secretion when compared to that of pyloric mucosa [118]. Additionally, the cellular localization of AQP4 is not only distributed in porcine enterocytes along the villi and crypt in the small and large intestines, but also in its duodenal Brunner’s glands and enteric neurons by immunohistochemistry [119]. Moreover, AQP8 mRNA is present in both the mucosa of jejunum and ileum in weaned piglets [95]. However, the expression of water channel proteins in different parts of the gastrointestinal tracts in domestic animals and their significance in gut physiology and pathology has yet to be elucidated.

## 5. Conclusions

The gastrointestinal tract, mainly the stomach and intestine discussed herein, is the major digestive and absorption site for nutrients and fluids that ensure normal gut functions. There is abundant water and fluid transferred through the epithelial cell layer in the stomach and intestine. The transepithelial fluid transport in the stomach and intestine may be mainly mediated by AQPs. Until now, at least 11 isoforms of AQPs (AQP1–11) have been shown to be present in the stomach, small intestine and large intestine. The current available data have indicated that the alterations of the expression and localization of these AQPs may be associated with the pathology of gastrointestinal disorders, such as gastritis, gastric cancer, enteritis, diarrhea, as well as colon and rectal cancers. On the one hand, the upregulation of the expression of AQP3 and AQP5 in the stomach indicates their important roles in the development of gastritis and gastric cancers. On the other hand, the downregulated expression of several AQPs (AQP1, AQP3, AQP4, AQP7, AQP8, AQP10 and AQP11) in the small and large intestines has been observed in the process of either enteritis or diarrhea. Although AQPs have been discovered for years, limited data could be found concerning the specific roles of each isoform in the stomach and intestine. The current review summarized the possibilities of AQPs as potential therapeutic targets in gastrointestinal disorders. Other important tissues in the gastrointestinal tract (such as salivary glands, liver, pancreas and gallbladder) are also very vital for gut functioning, and the roles of AQPs in these important tissues have been well summarized in recent reviews [17,120]. More investigations are required to elucidate the regulatory signal pathways for AQPs’ expression, localization and distribution changes in gut physiology and pathology. New research approaches, such as the CRISPR-Cas9 gene editing technique, the isotope labeling method and the Ussing chambers technique, can be applied in future studies to elucidate the roles and mechanism of specific AQPs in gut health and diseases.

## Figures and Tables

**Table 1:** Expression of aquaporins (AQPs) in the stomach of humans and rodents.

AQPs	Species	Distribution	Method	Reference
Human stomach				
AQP1	Human	Gastric mucosal tissues; stomach body; pyloric antrum	RT-PCR	[3,25,26]
AQP2	Human	Antral and oxyntic gastric mucosa	Tissue microarrays, IHC	[27]
AQP3	Human	Gastric antral and oxyntic mucosa; stomach body; pyloric antrum; MKN45 cells	RT-PCR, WB, tissue microarrays, IF	[3,25,27,29,30]
AQP4	Human	Gastric mucosal tissues; stomach body; MKN45 cells	RT-PCR, WB, IHC	[3,25,28,29,30]
AQP5	Human	Gastric mucosal tissues; stomach body; pyloric antrum; MKN45 cells	RT-PCR, WB, IHC, IF	[3,25,29]
AQP7	Human	Stomach body; pyloric antrum	RT-PCR	[3]
AQP8	Human	Stomach body	RT-PCR	[3]
AQP9	Human	Gastric gland and smooth muscle cells	IHC	^1^
AQP10	Human	Stomach body	RT-PCR	[3]
AQP11	Human	Gastric mucosal tissues; stomach antrum and body	RT-PCR	[3,25]
Murine stomach				
AQP1	Rat	Gastric mucosa	ELISA, IHC	[31]
AQP3	Rat	Gastric gland	RT-PCR, WB, IHC	[32]
AQP4	Rat, mouse, guinea pig	Parietal cells; gastric mucosa; gastric gland	RT-PCR, ELISA, WB, IHC, IF	[31,33,34,35,36,37]
AQP5	Rat	Pyloric gland	RT-PCR, WB, IHC, IF	[18,38]
AQP6	Rat	Gastric gland	RT-PCR, WB, IHC, IS	[19]
AQP8	Rat	Gastric gland	RT-PCR	[32]

^1^ Data from the website (Available online: http://www.proteinatlas.org/ENSG00000103569-AQP9/tissue/stomach); Abbreviations: IF, immunofluorescence; IHC, immunohistochemistry; IS, in situ hybridization; MKN45, human gastric adenocarcinoma; NB, Northern blotting; RT-PCR, reverse transcription-polymerase chain reaction; WB, Western blotting.

**Table 2:** Cellular localization of aquaporins (AQPs) in different cell types in stomach and intestines.

Tissue	Cell Types	Localization			Reference
		Apical	Intracellular	Basolateral	
Stomach	(1) Surface epithelial cells	AQP5	–	AQP3, AQP4	[3,13,31]
	(2) Gastric gland cells				
	Parietal cells	AQP5	–	AQP3, AQP4, AQP6	[11,13,19,33,36,37]
	Chief cells	AQP5	–	AQP4	[11]
	(3) Microvessels	AQP1	–	AQP1	[3,13]
Small intestine	(1) Villi epithelial cells	AQP6, AQP7, AQP10, AQP11 ?	AQP8	AQP3	[3,11,19,20,39]
	(2) Crypt epithelial cells	–	–	AQP3, AQP4	[3,11]
	(3) Goblet cells	AQP9, AQP10	–	AQP3	[39,40]
	(4) Paneth cells	–	–	AQP3	[39]
	(5) Microvessels	AQP1, AQP10	–	AQP1	[3,13,39]
Large intestine	(1) Villi epithelial cells	AQP2, AQP3, AQP7, AQP8, AQP10, AQP11 ?	AQP8	AQP3, AQP4	[3,11,21,41,42,43]
	(2) Crypt epithelial cells	AQP7, AQP8	AQP7, AQP8	–	[3,44]
	(3) Goblet cells	AQP9	–	–	[40]
	(4) Microvessels	AQP1	–	AQP1	[3,13]

–, not determined or not found; ?, unknown.

**Table 3:** Expression of aquaporins (AQPs) in the small intestine of human and murine rodents.

AQPs	Species		Distribution	Method	Reference
Human small intestine					
AQP1	Human	Duodenum, jejunum and ileum		RT-PCR, IHC	[21,66]
AQP2	Human	Duodenum, jejunum and ileum		Tissue microarrays, IHC	[27]
AQP3	Human	Duodenum, jejunum and ileum		RT-PCR, tissue microarrays, IHC	[21,27,66]
AQP4	Human	Duodenum, jejunum and ileum		RT-PCR	[66]
AQP7	Human	Ileum		RT-PCR, IHC	[21]
AQP8	Human	Ileum		RT-PCR, IHC	[21]
AQP10	Human	Duodenum, jejunum and ileum		RT-PCR, IS, NB, WB, IHC, IEM	[68,69]
AQP11	Human	Duodenum		RT-PCR	[3]
Murine small intestine					
AQP1	Rat, mouse	Duodenum, jejunum and ileum		RT-PCR, WB, IHC	[18,38,71,74]
AQP3	Rat, mouse	Jejunum and ileum		RT-PCR, WB, IHC	[32,71,73]
AQP4	Rat, guinea pig	Jejunum and ileum		WB, IF	[33,73]
AQP5	Rat	Duodenum		RT-PCR, WB, IF	[38]
AQP6	Rat	Duodenum, jejunum and ileum		RT-PCR, WB, IHC, IS	[19]
AQP7	Mouse, rat	Duodenum, jejunum and ileum		RT-PCR, WB, IHC	[14,71,75]
AQP8	Rat	Duodenum, jejunum and ileum		RT-PCR, WB, IHC	[32,75]
AQP9	Mouse	Duodenum, jejunum and ileum		RT-PCR, IS, WB	[40]
AQP11	Mouse	Jejunum and ileum		RT-PCR	[71]

Abbreviations: IEM, immunoelectron microscopy; IF, immunofluorescence; IHC, immunohistochemistry; IS, in situ hybridization; NB, Northern blotting; RT-PCR, reverse transcription-polymerase chain reaction; WB, Western blotting.

**Table 4:** Expression of aquaporins (AQPs) in the large intestine of human and murine rodents.

AQPs	Species	Distribution	Method	Reference
Human large intestine				
AQP1	Human	Colon	RT-PCR, WB, IF	[21]
AQP2	Human	Colon	Tissue microarrays, IHC	[27]
AQP3	Human	Colon	RT-PCR, WB, tissue microarrays, IHC	[21,27,42]
AQP4	Human	Colon	RT-PCR	[44]
AQP7	Human	Colon	RT-PCR, IHC	[21,44]
AQP8	Human	Colon	RT-PCR, IHC, NB, IS	[8,21,44]
AQP10	Human	Colon	RT-PCR	[3]
AQP11	Human	Colon	RT-PCR	[3]
Murine large intestine				
AQP1	Mouse	Colon	RT-PCR	[71]
AQP2	Rat	Colon	RT-PCR, WB, IS, IHC	[41,79]
AQP3	Rat, mouse	Colon and rectum	RT-PCR, WB, IHC	[32,78,80,81]
AQP4	Rat, mouse	Colon	RT-PCR, WB, IHC	[33,44,71,81]
AQP6	Rat	Colon and cecum	RT-PCR, WB, IHC, IS	[19]
AQP7	Mouse, rat	Colon, cecum, and rectum	WB, IHC	[14,44]
AQP8	Rat, mouse	Colon	RT-PCR, WB, IHC	[44,71,81]
AQP9	Mouse	Colon	IS	[40]
AQP11	Mouse	Colon	RT-PCR	[71]

Abbreviations: IF, immunofluorescence; IHC, immunohistochemistry; IS, in-situ hybridization; NB, Northern blotting; RT-PCR, reverse transcription-polymerase chain reaction; WB, Western blotting.

**Table 5:** Changes in the aquaporins (AQPs) during diarrhea in humans and animals.

Species	Cell Types	AQPs Changes	Level ^1^	Reference
*Escherichia coli*- and LPS-induced diarrhea				
Human	HT-29 cells	LPS decreased AQP3 expression	M, P	[92]
Rat	Colon	Inhibition of AQP3 function caused diarrhea	M, P	[93]
Mouse	Colon	AQP2 and AQP3 were mislocalized from cell membranes to the cytoplasm under EHEC and EPEC diarrhea	L	[83]
Piglet	Jejunum	ETEC challenge reduced mucosa AQP8 expression	M	[94]
Piglet	Jejunum	LPS challenge decreased mucosa AQP8 expression	M	[95]
Cholera toxin-induced secretory diarrhea				
Human	Duodenum	AQP10 expression was downregulated	M	[96]
Rat	Small intestine	Mucosal AQP8 expression was decreased	M	[97]
Allergy-induced diarrhea				
Human	Colon	AQP3, AQP7, AQP10 and AQP11 expression was decreased in celiac disease patients	M, P	[3,22]
Mouse	Proximal colon	AQP4 and AQP8 were downregulation when there was an allergy	M, P	[98]
Drug-induced diarrhea				
Mouse	Colon	AQP1 and AQP11 expression was decreased in 5-fluorouracil-induced diarrhea	M	[71]
Mouse	Colon	AQP4 and AQP8 expression was decreased in 5-fluorouracil-induced diarrhea	M, P	[71]
Rotavirus diarrhea				
Mouse	Colon	AQP1, AQP4 and AQP8 expression was downregulated in rotavirus diarrhea	P	[99]

^1^ M, mRNA expression level; P, protein expression level; L, localization changes. Abbreviations: EHEC, enterohemorrhagic *Escherichia coli*; EPEC, enteropathogenic *Escherichia coli*; ETEC, enterotoxigenic *Escherichia coli*; HT-29, human colon epithelial cells; LPS, lipopolysaccharide.
